# Supplementary material for: Exploring Doctors’ Emerging Commitment to Rural and General Practice Roles over Their Early Career
Source: Int J Environ Res Public Health. 2021 Nov 11;18(22):11835. doi: 10.3390/ijerph182211835 (PMC8619547; doi:10.3390/ijerph182211835)
Supplement: Supplementary file 1 [file ijerph-18-11835-s001.zip › ijerph-1414055-supplementary.pdf]

Supplementary Materials

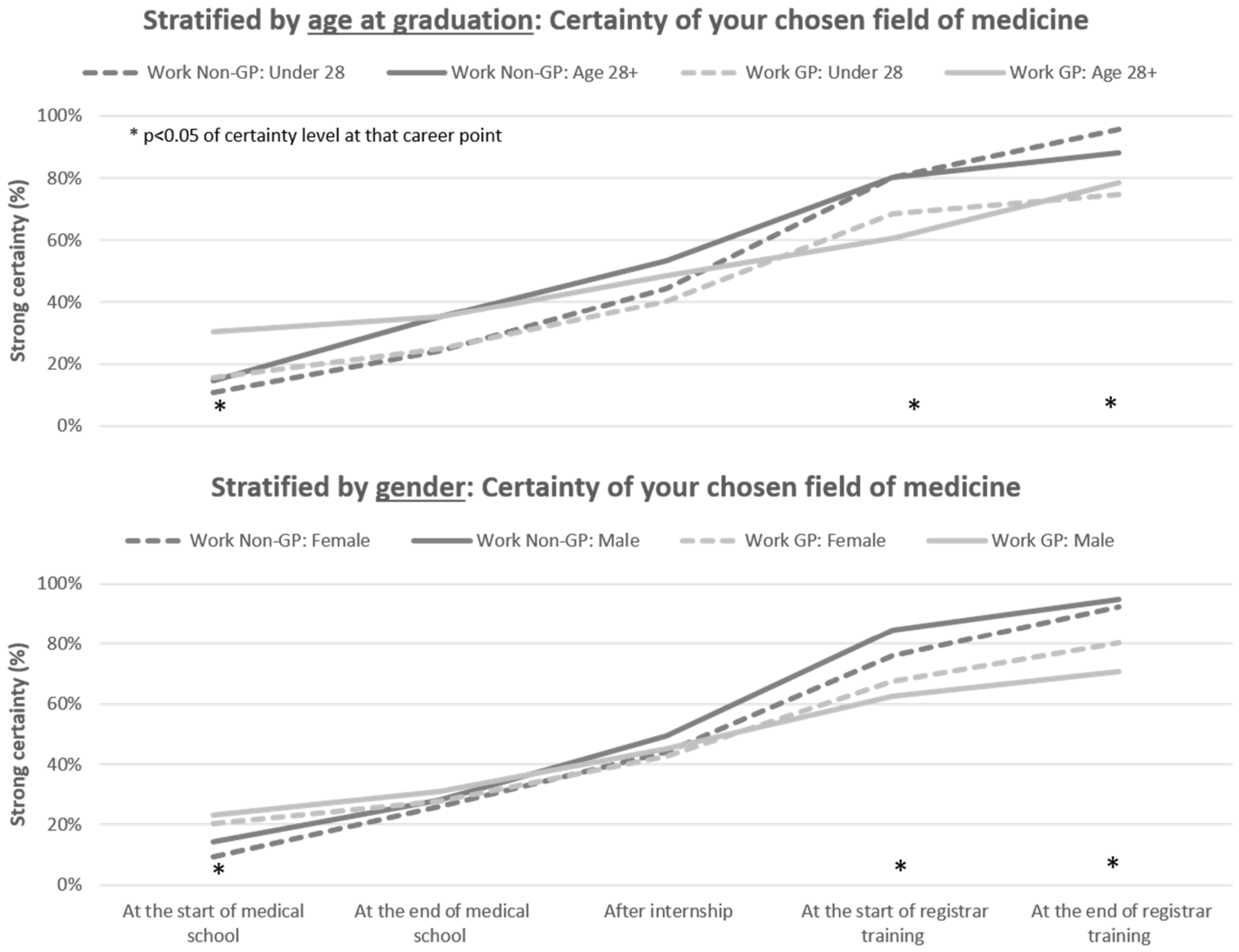

Figure S1. Level of certainty of their specialty decision amongst junior doctors, by other key strata (age at graduation, gender).

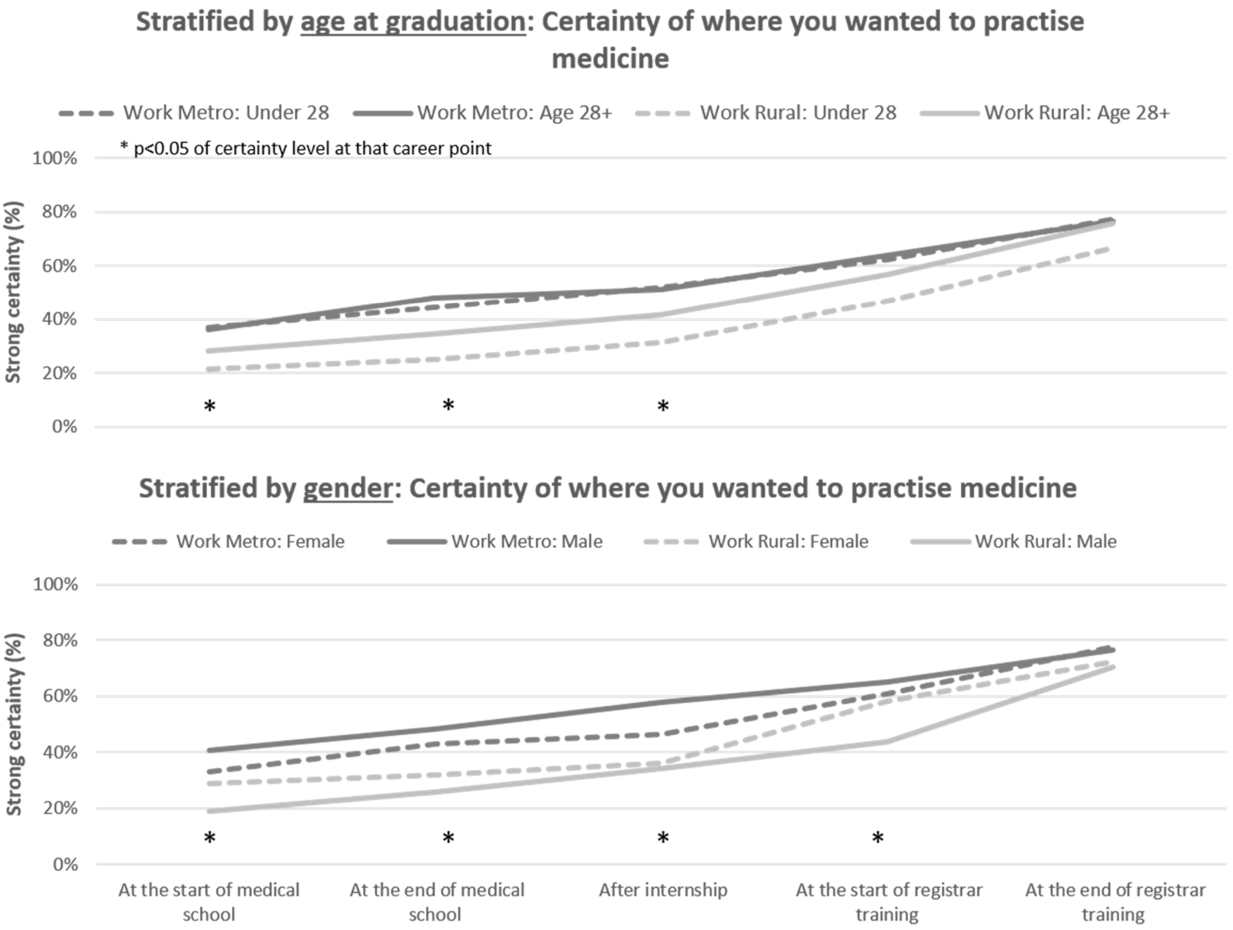

Figure S2. Level of certainty of their practice location decision amongst junior doctors, by other key strata (age at graduation, gender).
